# Changes in pregnancy outcomes during the COVID-19 lockdown in Iran

**DOI:** 10.1186/s12884-021-04050-7

**Published:** 2021-08-22

**Authors:** Fahimeh Ranjbar, Leila Allahqoli, Soheila Ahmadi, Robab Mousavi, Maryam Gharacheh, Nooshin Eshraghi, Ibrahim Alkatout

**Affiliations:** 1 Reproductive Health, Nursing Care Research Centre, Iran University of Medical Sciences, P.O. Box 19395-4798, Rashid Yasemi St., Valiasr Ave., Tehran, Iran; 2 School of Nursing and Midwifery, Iran University of Medical Sciences, Tehran, Iran; 3 Department of Perinatology, Shahid Akbarabadi hospital, School of Medicine, Iran University of Medical Sciences, Tehran, Iran; 4 Department of Obstetrics and Gynecology, Kiel School of Gynaecological Endoscopy. Campus Kiel, University Hospitals Schleswig-Holstein, Kiel, Germany

**Keywords:** Covid-19 pandemic, Maternal outcomes, Neonatal outcomes

## Abstract

**Background:**

The Covid-19 pandemic response is influencing maternal and neonatal health care services especially in developing countries. However, the indirect effects of Covid-19 on pregnancy outcomes remain unknown. The aim of the present study was to compare pregnancy outcomes before and after the beginning of the Covid-19 pandemic in Iran.

**Methods:**

We performed a retrospective analysis of the medical records of 2,503 pregnant women with singleton pregnancies, admitted to the maternity department of a women’s hospital in Tehran, Iran, during the pre-Covid-19 pandemic (February 19 to April 19, 2019) and the intra-Covid- 19 pandemic (February 19 to April 19, 2020) period.

**Results:**

We included 2,503 women admitted to the hospital; 1,287 (51.4 %) were admitted before the Covid-19 lockdown and 1,216 (48.6 %) during the Covid-19 lockdown. There were no significant differences in stillbirth rates (*p* = 0.584) or pregnancy complications (including preeclampsia, pregnancy-induced hypertension and gestational diabetes) (*p* = 0.115) between pregnant women in the pre- and intra-pandemic periods. However, decreases in preterm births (*p* = 0.001), and low birth weight (*p* = 0.005) were observed in the pandemic period compared to the pre-pandemic period. No significant difference in the mode of delivery, and no maternal deaths were observed during the two time periods.

**Conclusions:**

In our study we observed a decrease in preterm births and low birth weight, no change in stillbirths, and a rise in the admission rates of mothers to the ICU during the initial Covid-19 lockdown period compared to pre-Covid-19 lockdown period. Further research will be needed to devise plan for immediate post-pandemic care and future health care crises.

## Background

Coronavirus infection (Covid-19) is a global public health emergency [1] and an emerging disease with a rapid increase in prevalence rates and deaths, causing illness ranging from the common cold to severe and fatal illness [2]. According to the WHO report, as of 29 December 2020, global cumulative numbers have risen to more than 79 million reported cases and over 1.7 million deaths since the beginning of the pandemic [3]. In Iran, there were 3,394,279 confirmed cases of Covid-19 from 3 to 2020 to 13 July 2021, with 86,041 deaths, reported to the WHO [4]. The disease is particularly lethal in susceptible populations.

Due to physiological changes in their cardiopulmonary and immune systems, pregnant women are more likely to manifest severe illness after infection with respiratory viruses [5]. Although the majority of coronavirus infections are mild, the severe acute respiratory syndrome coronavirus (SARS-CoV) and the Middle East respiratory syndrome coronavirus (MERS-CoV) epidemics of the recent decades have been serious; about a third of pregnant women who acquired the disease died from the infection (in: [6]). The effect of a Covid-19 infection on pregnant women appears to be less severe [5]. In a recent study by Chen et al. comprising nine women with Covid-19 in the third trimester of pregnancy, pregnancy complications that appeared after the onset of the Covid-19 infection were fetal distress and premature rupture of the membrane (in two of nine). All of the women underwent caesarean section; no woman experienced severe Covid-19 pneumonia and no deaths were encountered [7].

The burden of the Covid-19 pandemic on health care institutions has led to growing concerns about the disruption of health services. Disruption of maternal and neonatal health services could result in adverse birth outcomes, especially in developing countries [8]. Currently, data on the effect of Covid-19 on pregnancy outcomes is limited [5]. A systematic review showed that pregnant women with Covid-19 had higher rates of preterm birth, preeclampsia, cesarean delivery, and perinatal death compared to the general population [9]. An increase in stillbirths was reported during the Covid-19 pandemic in the UK, which was unlikely to have been caused directly by viral infection because none of the patients had Covid-19. This observation may be explained by the reluctance of pregnant women to visit a hospital due to their fear of contracting SARS-CoV-2, or changes in obstetric services [10]. Previous public health crises have shown that the effect of an epidemic on maternal health is frequently not identified. The reason is that the impacts are not the direct consequence of the disease, but are caused indirectly by service interruptions and redirected resources [11]. The outbreak of the Ebola virus disease [12] caused delays in the care of women, which led to adverse maternal health outcomes [13]. Such scenarios may be expected during the Covid-19 pandemic as well. Pregnant women are at high risk for the infection [14] and need special attention as regards prevention, diagnosis, and treatment [5].

Based on the above mentioned studies, no conclusive statements can be made yet about the impact of the Covid-19 pandemic on maternal and fetal health. There is an urgent need to assess care-seeking behavior, maternity service delivery, and pregnancy outcomes in order to plan for immediate post-pandemic care as well as future health system crises [10]. The aim of the present study was to compare pregnancy outcomes before and after the beginning of the Covid-19 pandemic in Iran. The results are expected to assist in improving the care and management of pregnant women and infants during pandemics, and prevent adverse maternal and child health outcomes.

## Methods

This is a retrospective analysis of the medical records of 2,503 pregnant women with singleton pregnancies, admitted to the maternity department of Akbarabadi hospital (a women’s hospital in Tehran, Iran) during the pre-Covid-19 pandemic period (February 19 to April 19, 2019) and Intra-Covid-19 pandemic period (February 19 to April 19, 2020). The time period covered the first wave of the pandemic and the subsequent lockdown.

Data on maternal characteristics (such as maternal age, gestational age, parity, gravidity, abortion, living children and the number of prenatal care visits) and pregnancy outcomes were entered in a data form. Pregnancy outcomes in the two time periods were compared. Pregnancy outcomes included maternal health outcomes (such as preterm birth, miscarriage, antepartum hemorrhage, caesarean section, and maternal death) and neonatal health outcomes (including low birth weight, Apgar score, stillbirths, and the NICU admission rate). Pregnancy complications such as gestational diabetes, pregnancy-induced hypertension and preeclampsia, were also compared in the two time periods. Preterm birth was defined as birth after < 37 weeks of gestation, based on the first day of the woman’s last menstrual period. A miscarriage was defined as a loss of pregnancy during the first 22 weeks of gestation. Maternal death referred to deaths due to complications from pregnancy or childbirth. A stillbirth was defined as a baby being born dead after 22 weeks of gestation, and low birth weight was defined as a birth weight less than 2500 g.

### Statistical analysis

Data were analyzed using the SPSS software version 22. Comparisons between pre- and intra-Covid-19 pandemic periods were focused on two months of the same period of the year in order to take the potential effect of seasonal differences into account. Qualitative variables were described by frequency (percentage) and compared between the two time periods with the Chi-square test. Quantitative variables were expressed in means and standard deviation (SD), and compared by the independent samples t test. Normal distribution of data was assessed using a one–sample Kolmogorov-Smirnov test. A *p*-value less than 0.05 was considered statistically significant.

## Results

We included 2,503 women admitted to the hospital, of whom 1,287(51.4 %) were admitted before the Covid-19 lockdown and 1,216 (48.6 %) during the lockdown. The mean age of women during the pandemic was significantly lower than that of women in the pre-pandemic period. The numbers of pregnancies (*p* = 0.005), deliveries (*p* = 0.001), living children (*p* = 0.001), and prenatal care visits (*p* = 0.001) during the intra-pandemic period were higher than those in the pre-pandemic period. Maternal characteristics between the pre- and intra-pandemic periods are summarized in Table 1.

**Table 1:** Comparison of maternal characteristics in the pre- and intra-pandemic periods

	Pre-pandemic period *n* = 1287	Intra-pandemic period *n* = 1216	Total *n* = 2503	*p*-value
Mean maternal age (years)	29.22 ± 6.54	28.23 ± 6.17	28.73 ± 6.38	t = 3.93 df = 2497 *p* = 0.001
Mean gestational age (weeks)	35.52 ± 7.64	35.88 ± 7.38	35.70 ± 7.52	t = 1.196 df = 2501 *p* = 0.232
Gravidity (mean)	2.12 ± 1.28	2.29 ± 1.73	2.20 ± 1.51	t = 2.839 df = 2474 *p* = 0.005
Parity	1.63 ± 0.89	1.82 ± 1.08	1.72 ± 0.99	t = 3.695 df = 1510 *p* = 0.001
Abortions (mean)	0.35 ± 0.76	0.36 ± 0.69	0.35 ± 0.73	t = 0.326 df = 2466 *p* = 0.744
Living children (mean)	1.61 ± 0.86	1.77 ± 1.05	1.7169 ± 0.96	t = 4.199 df = 2466 *p* = 0.001
Mean numbers of prenatal care visits	6.63 ± 2.09	7.05 ± 1.85	6.89 ± 1.96	t = 3.224 df = 737.24 *p* = 0.001

There were no significant differences in pregnancy complications (including preeclampsia, pregnancy-induced hypertension, and gestational diabetes; *p* = 0.115) between the pre- and intra-pandemic periods. Preterm birth (*p* = 0.001) and low birth weight (*p* = 0.005) rates were lower in the pandemic period than in the pre-pandemic period. However, a significant increase in the maternal admission rates to the ICU (3.6 % vs. 6.6 %, *p* = 0.001) for medical reasons was observed during the pandemic period. No significant difference was noted in the mode of delivery (Table 2) and no maternal death was encountered in the two time periods.

**Table 2:** Comparison of pregnancy outcomes in the pre- and intra-pandemic periods

Pregnancy outcomes		Pre-pandemic period *n* = 1287	Intra-pandemic period *n* = 1216	Total *n* = 2503	*p*-value
Antepartum hemorrhage	No	1144 (96.5 %)	1152 (97.8 %)	2296 (97.2 %)	χ²=3.365 df = 1 *p* = 0.067
	Yes	41 (3.5 %)	26 (2.2 %)	67 (2.8 %)	
Preterm births	No	894 (76.8 %)	932 (83.1 %)	1826 (79.9 %)	χ²=13.937 df = 1 p = 0.001
	Yes	270 (23.2 %)	190 (16.9 %)	460 (20.1 %)	
Mode of delivery	NVD	539 (45.8 %)	562 (49.7 %)	1101 (47.7 %)	χ²=3.66 df = 1 *p* = 0.056
	CS	639 (54.2 %)	568 (50.3 %)	1207 (52.3 %)	
Admission of mothers to the ICU	No	1241 (96.4 %)	1134 (93.4 %)	2375 (95 %)	χ²=11.875 df = 1 p = 0.001
	Yes	46 (3.6 %)	80 (6.6 %)	126 (5 %)	
Admission of neonates to the NICU	No	859 (72.9 %)	909 (80.6 %)	1768 (76.7 %)	χ²=18.926 df = 1 *p* = 0.001
	Yes	319 (27.1 %)	219 (19.4 %)	538 (23.3 %)	
Low birth weight	No	956 (83.9 %)	948 (88.0 %)	1904 (85.9 %)	χ²=7.914 df = 1 *p* = 0.005
	Yes	184 (16.1 %)	129 (12.0 %)	313 (14.1 %)	
Stillbirths	No	1144 (97.1 %)	1091 (96.7 %)	2235 (96.9 %)	χ²=0.30 df = 1 *p* = 0.584
	Yes	34 (2.9 %)	37 (3.3 %)	71 (3.1 %)	

The birth weight of neonates born during the pandemic was higher than that of neonates born in the pre-pandemic period (*p* = 0.009). The NICU admission rate (19.4 % vs. 27.1 %, *p* = 0.001) was lower during the pandemic compared to the pre-pandemic period. There was no significant difference in stillbirth rates (*P* = 0.584) between the two time periods.

During the Covid-19 lockdown, six patients with Covid-19 were hospitalized in the maternity department, with a mean gestational age of 36.50 ± 3.10 weeks. Three of the patients underwent a caesarean section in their third trimester, two patients presented with preeclampsia, and one was hospitalized in the ICU. No furtherpregnancy complications, maternal deaths or neonatal deaths occurred in these patients. Three of six newborns were born preterm, two had a low birth weight and three were hospitalized in the NICU. None of them was infected with Covid-19.

## Discussion

Measures to curtail the spread of SARS-CoV‐2 infection in Iran were initiated on February 19, 2020. To the best of our knowledge, this is the first study to focus on the indirect impact of the Covid-19 lockdown on maternal and neonatal outcomes in Iran. The investigation revealed a reduction in preterm births and low birth weight during the lockdown compared to the pre-lockdown period. The Covid-19 lockdown has considerably changed our lives in terms of work environments, reduced social interaction, and greater focus on hygiene. This situation may have influenced pregnancy outcomes [15]. Recent studies showed a decline in preterm birth rates [16, 17] and low birth weight [18] during the Covid-19 pandemic compared to the preceding time periods. In contrast, Wood et al. observed no reduction in preterm birth rates [19]. Other studies reported no changes during the lockdown period [20, 15, 21, 22]. Different approaches to birth outcomes research might explain the differences between studies. Moreover, the implementation of Covid-19 mitigation measures and population responses, as well as risk factors for adverse pregnancy outcomes differ from one country to another.

The lockdown in Iran, in response to the pandemic included the cancelation of public events, closure of schools, universities, shopping centers, holy shrines, and a ban on festival celebrations. Further measures included outposts for identifying Covid-19 at city entrances and screening through a hotline for detecting Covid-19 and providing relevant health information [23]. Similar to studies that reported a lower rate of adverse pregnancy outcomes, our findings in regard of reduced preterm births and low birth weight during the lockdown may be explained by a variety of factors. The latter include greater focus on hygiene and home confinement, less work-related strain, more opportunities for rest and nutritional support, the support systems provided during the lockdown, reduced exposure to infection [18, 21] as well as the postponement or suspension of medical interventions leading to iatrogenic preterm delivery. According to Phillip et al. the Covid-19 lockdown was likely to cause socio-environmental changes and behavioral modifications, and thus exert a beneficial impact on pregnancies during this period [18]. We observed a rise in the number of prenatal care visits during the lockdown. Evidence suggests a link between the adequacy of prenatal care and birth outcomes [24–27]. In a longitudinal study in Sweden, health-related worries were higher during the Covid-19 pandemic [28]. Women were more likely to attend healthcare centers for prenatal care in order to reduce their health concerns. Although the Covid-19 pandemic has had considerable effects on the coverage and quality of healthcare for mothers and newborns [29], the delivery of prenatal care did not change during the Covid-19 lockdown in Iran.

Our data showed no change in stillbirths during the lockdown. A nationwide cohort study in Sweden [20] and a retrospective study in Spain [21] also reported no change in stillbirth rates among the different study periods. In contrast, Ashish et al. in Nepal [8] and Khalil et al. in the UK [10] reported a significantly higher rate of stillbirth during the pandemic compared to the pre-pandemic period. The authors discussed that the rise in stillbirths may have resulted from indirect effects such as less frequent use of health facilities due to fear of contracting infection as well as changes in health care delivery. Small sample sizes and single-center designs may be sensitive to random variations [20], and may explain the different results in the studies.

Our investigation revealed similar rates of caesarean section during the pre-pandemic period and the lockdown, but mothers were admitted more frequently to the ICU during the lockdown. Goyal et al. also reported a higher rate of ICU admissions for pregnant women during the pandemic compared to the pre-pandemic period [30]. Higher admission rates to the ICU do not appear to be a direct consequence of Covid-19 infection, but could be attributed to a rise in comorbidities and underlying diseases due to the reluctance of high-risk mothers to seek care in hospitals during the lockdown. According to Ashish et al., women with a high risk of complications did not seek care at healthcare facilities regularly, and complications resulted from delayed care and other factors associated with the lockdown [8].

Evidence suggests that infection with SARS-CoV2 does not affect the mode of delivery [31, 32]. However, a half of the women with Covid-19 in our study underwent a cesarean section and one third presented with preeclampsia. One half of the patients had preterm births and one third of the newborns had a birth weight of less than 2500 g. A meta-analysis showed that Covid-19 infection was associated with higher rates of cesarean section, preterm birth, preeclampsia, and perinatal mortality [9]. However, in a retrospective study performed by Zhang et al. in Hubei province, China, rates of preterm birth and neonatal asphyxia did not differ between pregnant women with a Covid-19 infection and controls [33]. Women with the most serious Covid-19 infection were those with the highest rates of comorbidities such as diabetes, gestational hypertension or preeclampsia [34], which may lead to adverse pregnancy outcomes. Although there is limited evidence of intrauterine vertical transmission of Covid-19, infected pregnant mothers may be subject to a high risk of severe respiratory complications [35]. Similar to previous reports, in our study all pregnant women with Covid-19 were admitted to the hospital in the third trimester. We were able to confirm the pattern observed for other respiratory viruses: women in a later phase of pregnancy are more severely affected by the respective disease [36].

We found that, compared to the pre-lockdown period, women during the lockdown were younger and had higher rates of parity and previous pregnancies. Notwithstanding the precautions taken to curtail the coronavirus disease in Iran, the authorities had to deal with numerous challenges in combating the pandemic, including a shortage of personal protective equipment such as masks, disinfectants and hospital gowns, inadequate numbers of hospital beds and equipment and shortage of health care workers [23]. Therefore, Covid-related restrictions may have caused mothers from a high socioeconomic level to seek care in private hospitals for fear of the disease and concerns about poor facilities in public hospitals. These women are most likely to have a higher maternal age and lower parity and gravidity rates. This may explain the higher parity and gravidity rates as well as the higher birth weight observed during the lockdown compared to the pre-lockdown period. Demographic differences between the two groups may also have influenced the findings and must be kept in mind as a limitation of the study.

In the present study, data on pregnancy outcomes were obtained from a single women’s referral hospital in Tehran, and were derived from the women’s medical records in two time periods of two months each. The retrospective nature of the study and its short time frame are major limitations. Besides, we did not assess the direct impact of Covid-19 on pregnancy outcomes.

## Conclusions

The present study revealed a decrease in preterm birth rates and low birth weight, no change in stillbirths, and a rise in maternal ICU admission rates during the initial Covid-19 lockdown period. A lesson learned from the lockdown is that doctors possibly medicalize a large number of pregnancies and perform iatrogenic preterm deliveries. However, the link between pregnancy outcomes and the lockdown remains ambiguous. Further research will be needed to gain more knowledge about numerous ways in which social, environmental, and behavioral factors influence pregnancy outcomes. There is also a need to assess maternal and neonatal health service delivery. Research in these areas will enable us to draft plans for immediate post-pandemic care and future health system crises, and allocate resources effectively.

## Data Availability

The data that support the findings of this study are available from the research deputy of Iran University of Medical Sciences but restrictions apply to the availability of these data, which were used under license for the current study, and so are not publicly available. Data are however available from the corresponding author upon reasonable request and with the permission of research deputy of Iran University of Medical Sciences. You can direct correspondence to gharacheh.m@gmail.com.

## Abbreviations

COVID-19: Coronavirus Disease 2019
ICU: Intensive care unit
MERS-CoV: Middle East respiratory syndrome coronavirus
NICU: Neonatal intensive care unit
SARS-CoV: Severe acute respiratory syndrome coronavirus
